# Decisions on eating and drinking in older adults admitted with pneumonia and referred for swallowing difficulties

**DOI:** 10.1007/s41999-024-00983-2

**Published:** 2024-05-09

**Authors:** Yuki Yoshimatsu, Dharinee Hansjee, Marianne Markowski, Ryan Essex, David G. Smithard

**Affiliations:** 1 Elderly Care, Queen Elizabeth Hospital, Lewisham and Greenwich NHS Trust, Stadium Rd, London, SE18 4QH UK; 2 Centre for Exercise Activity and Rehabilitation, School of Human Sciences, University of Greenwich, London, UK; 3 Speech and Language Therapy, School of Health Sciences, University of Greenwich, London, UK; 4 The Institute for Lifecourse Development, University of Greenwich, London, UK

**Keywords:** Dysphagia, Aspiration pneumonia, Choking, Risk feeding, Comfort feeding, Modified diet

## Abstract

**Purpose:**

Older patients with pneumonia are commonly restricted from oral intake due to concerns towards aspiration. Eating and drinking with acknowledged risks (EDAR) is a shared decision-making process emphasising patient comfort. As part of our project to find the barriers and facilitators of EDAR, we aimed for this initial study to see how frequently EDAR was selected in practice.

**Methods:**

We performed a retrospective cohort study at an acute hospital where EDAR was initially developed, of patients aged ≥ 75 years-old admitted with pneumonia and referred to speech and language therapy.

**Results:**

Out of 216 patients, EDAR decisions were made in 14.4%. The EDAR group had a higher 1-year mortality than the modified/normal diet groups (*p* < 0.001). Pneumonia recurrence rate did not differ significantly between the groups (*p* = 0.070).

**Conclusion:**

EDAR decisions were comparatively less common and most were associated with end-of-life care. Underlying reasons for the low EDAR application rate must be investigated to maximise patient autonomy and comfort as intended by EDAR while minimising staff burden.

## Key summary points

**Aim:**

We examined the frequency of different decisions, including eating and drinking with acknowledged risks (EDAR) in a single-institution retrospective study of older people with pneumonia and swallowing difficulties.

**Findings:**

EDAR decisions were made in only a small fraction of patients (less than one fourth of patients on a modified diet). Most EDAR decisions were for end-of-life comfort care, and patients for EDAR had a significantly higher mortality despite the pneumonia recurrence rate not differing significantly.

**Message:**

The reasons underlying the relatively low frequency of EDAR decisions compared to modified diet needs to be investigated to maximise patient autonomy and comfort while minimising staff burden.

## Introduction

When a frail older adult is admitted to the hospital with pneumonia, the aetiology is frequently attributed to aspiration[1, 2]. When aspiration is suggested, clinicians frequently restrict the patient from eating and drinking until assessed by a speech and language therapist (SLT). The SLT will advise on the patient’s ability to swallow safely. The management plan will vary from a normal diet (ND), through a modified diet (MD), or suggestion that the patient is too unsafe to eat and drink at all. Modified diet and nil-by-mouth (NBM) orders are associated with dehydration, malnutrition, oral health decline, poor quality of life and increased mortality[3].

For some patients, a better approach is to support them to eat and drink despite the risks; this is often termed “Risk Feeding” or “Eating and Drinking with Acknowledged Risks (EDAR).” EDAR is an alternative shared decision-making process that enables comfort, dignity, and autonomy for patients who prefer to continue oral intake, or where alternative management strategies such as tube feeding are inappropriate. In recent years, guidance has been developed by the Royal College of Speech and Language Therapists (RCSLT) to assist the decision-making process[4]. The recommended EDAR decision-making process includes a capacity assessment, a clinical evaluation of the swallow, establishing the goal of care, facilitating communication within the multidisciplinary team, and setting out an advance care plan where appropriate[4]. While the initial idea of EDAR may be suggested by the SLT, it is a patient-led decision. Capacity assessment forms part of the decision-making process, and the patient is always involved if they are capable. The Royal College of Physicians (RCP) has also published guidance on supporting people with eating and drinking difficulties[5].

However, questions have been raised regarding the risk management approach of the RCP guidance[6]. Moreover, despite guidance being available, in the clinical setting, supporting patients’ choices (or identifying patients who would benefit from EDAR even when their choice is unclear) and making these complex decisions remain a medical and ethical struggle. It is important to investigate how EDAR decisions are made in daily practice, to consider the next steps in further promoting it for appropriate patients.

We therefore conducted a retrospective study on how EDAR decisions are made in daily clinical practice in the management of older adults in hospital with a diagnosis of community-acquired pneumonia (CAP).

## Methods

We performed a retrospective cohort study of older patients admitted with a diagnosis of pneumonia to Queen Elizabeth Hospital (Lewisham and Greenwich NHS Trust). Ethical approval was obtained from the Lewisham and Greenwich NHS Trust (Number 7211), and informed consent was waived due to the retrospective nature of the study.

We included patients aged 75 years-old and above admitted to the hospital with a diagnosis of CAP from 1st January 2021 to 31st December 2021 and were referred to an SLT for the assessment of suspected swallowing impairment. We excluded those who were admitted for COVID-19 pneumonitis, those who were admitted for more than once during the study period (only the first admission was included), those who did not have pneumonia according to the medical records, those who developed pneumonia after admission, and those admitted with a hospital acquired pneumonia.

We divided the patients into four groups according to the initial decisions made regarding their oral intake: the ND group, MD group, EDAR group, and NBM group. We compared the following between the four groups: patient backgrounds (age, Rockwood Clinical Frailty Scale (CFS)[7], initial diagnosis made by the consultant (aspiration pneumonia or non-aspiration pneumonia), pneumonia severity index (PSI)[8] and outcomes (in-hospital and 1-year mortality, pneumonia recurrence within 30 days). For the EDAR group, the reason for selecting EDAR was also extracted.

### Statistical analyses

We used chi-square tests to compare outcomes and the one-way ANOVA test for continuous parametric variables (age, CFS and PSI). Analyses were performed using Microsoft Excel and online resources[9]. A *p* value < 0.05 was considered to be statistically significant for all analyses. Post hoc tests were performed where initial results indicated significant differences.

## Results

**Fig. 1:**
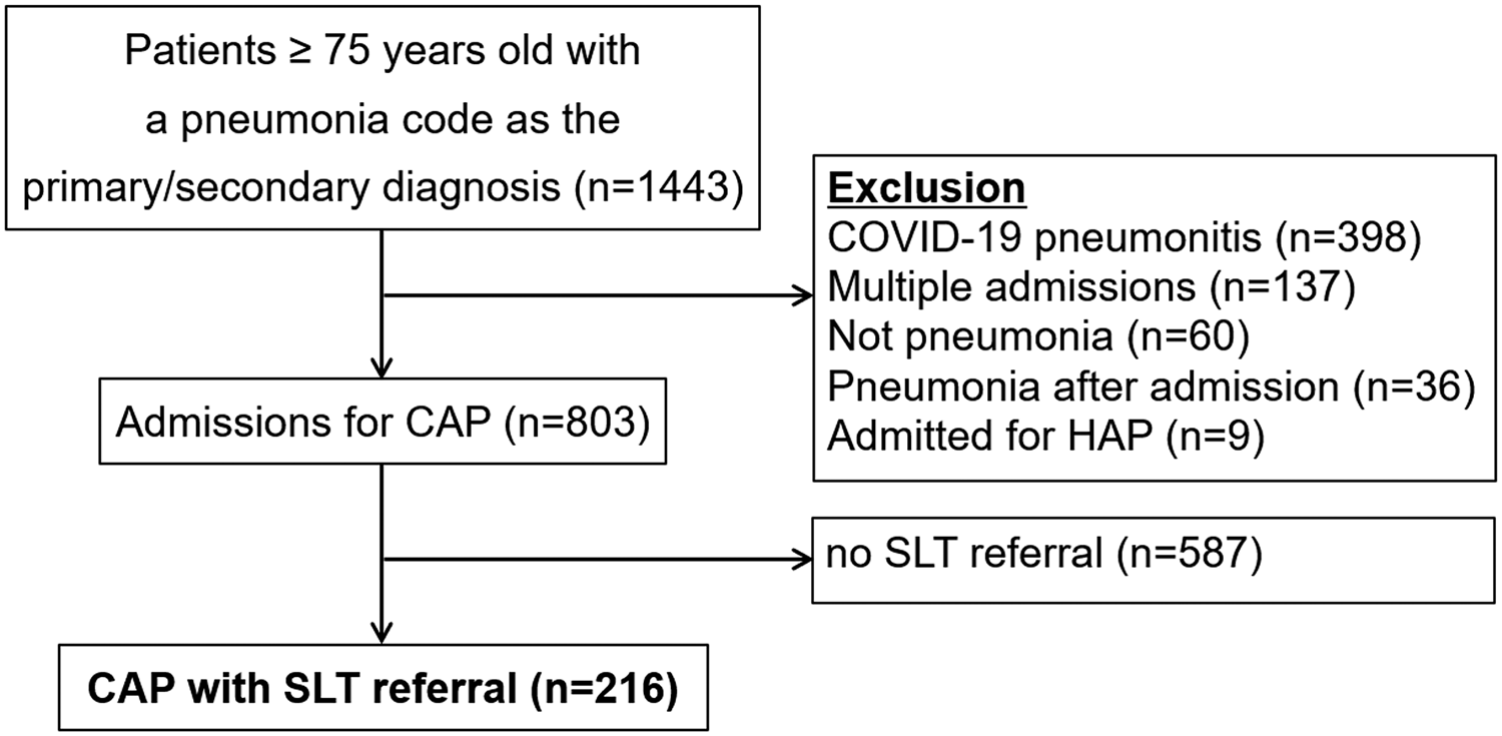
Patient selection. *CAP* community-acquired pneumonia, *HAP* hospital-acquired pneumonia, *SLT* speech and language therapist

The initial list of 803 patients aged 75 years-old and above admitted with a diagnosis of CAP had a median age of 84 years-old (interquartile range 80–89) and a CFS score of 5 (4–6). 216 patients who underwent SLT assessment were included in the study (Fig. 1). Of these patients, 14.4% were considered appropriate for EDAR, 59.3% for MD, 19.9% for ND, and 6.5% for NBM. Demographic data and outcomes are summarised in Table 1. Of the 31 patients who were eating and drinking with acknowledged risks, the reasons underlying the decisions were short life expectancy (58.1%), quality of life (38.7%), and refusing nasogastric tube feeding (3.2%). Only 19.4% of these patients were assessed as having the mental capacity to make these decisions. For those without capacity, attempts were made by the team to establish the wishes of the patient from significant others which forms part of the decision-making process. The EDAR decisions were mostly initiated by the SLT following a swallow assessment and then discussed with the doctor, patient (when having capacity), and family member. A shared decision making process was co-ordinated by SLT to ensure the patient’s views are included as part of the MDT decision.

**Table 1:** Characteristics and outcomes of patients admitted with pneumonia

	ND (*n* = 43)	MD *(n* = 128)	EDAR (*n* = 31)	NBM (*n* = 14)	*p* value
Characteristics					
Age: median (IQR)	86 (81, 90)	85 (80, 91)	89 (84, 94)	85 (82, 92)	0.087
Male sex	44.2%	53.9%	35.5%	50.0%	0.272
CFS: median (IQR)	6 (4, 7)	6 (5, 7)	7 (5, 7)	5 (5, 6)	0.007
PSI: median (IQR)	100 (80, 122)	112 (99, 128)	122 (104, 132)	105 (95, 119)	0.162
Initial diagnosis AP	23.3%	44.5%	67.7%	50.0%	0.002
Outcomes					
Mortality, in hospital	12 (27.9%)	20 (15.6%)	15 (48.4%)	11 (78.6%)	< 0.001
Mortality, 1 year	26 (60.5%)	81 (63.3%)	29 (93.5%)	14 (100%)	< 0.001
Recurrence, 30 days	5/28 (17.9%)	21/100 (21.0%)	5/9 (55.6%)	0/3 (0%)	0.070

*ND* normal diet, *MD* modified diet, *EDAR* eating and drinking with acknowledged risks, *NBM* nil by mouth, *IQR* interquartile range, *CFS* clinical frailty scale, *PSI* pneumonia severity index, *AP* aspiration pneumonia

### Patient background

The patients included in the study had a median age of 86 years-old (interquartile range: 81–91). As shown in Table 1, significant differences among groups were indicated for frailty and being diagnosed with aspiration pneumonia. Post-hoc Tukey’s test revealed a statistically significant difference in CFS between the EDAR and ND groups (F(3212) = 4.14, *p* = 0.010) but not among any other groups. Post hoc comparison with Bonferroni correlation (adjusted alpha = 0.00625) indicated that an aspiration pneumonia diagnosis was significantly more common in the EDAR group than the ND group (*p* < 0.001) but not among any other groups.

### Outcomes

The EDAR and NBM groups showed a high short/long-term mortality, with half dying during the hospital stay and over 90% dying within a year. Bonferroni correlation (adjusted alpha = 0.00625) indicated that in-hospital mortality was significantly higher in the NBM group than in each of the three other groups (*p* < 0.001), but there were no significant differences among other groups. One-year mortality was significantly higher in the EDAR group compared to the ND group (*p* = 0.001) and MD group (*p* = 0.001), and in the NBM group compared to the ND group (*p* < 0.001) but not with any other groups. The pneumonia recurrence rate within 30 days did not differ significantly among the groups (*p* = 0.070), as shown in Table 1.

## Discussion

Our study revealed how EDAR decisions were not common in older patients diagnosed with pneumonia; EDAR decisions were made for one-fourth of patients compared to those offered MD alone. Reasons for this may include patient choice, physical condition, staff anxiety towards potentially contributing to risks of pneumonia and patient discomfort, staff members’ lack of awareness/understanding on EDAR, or staff members understanding but not wanting to support EDAR. Despite the setting being where EDAR was originally developed[10], there may still be a degree of insufficient awareness and understanding of EDAR. This was implied by the data that EDAR was chosen in more frail patients with higher severity of pneumonia, with the majority being chosen for end-of-life comfort care rather than a way to continue oral intake in patients with treatable pneumonia. This indicates a necessity for continuous education and training in the workplace. Choices and preferences, which form the foundation of EDAR decisions are not merely a part of terminal care but is also integral in the acute stages of disease. EDAR was established to enable patients the choice to continue oral intake regardless of disease stage, particularly where the patient refuses to accept modified food and liquids. It may be important at this stage to reconsider how and to whom to offer EDAR as a viable option.

The prognosis of older adults diagnosed with pneumonia (aspiration pneumonia in particular) is considerably poor[11, 12], and multimodal multidisciplinary care is imperative[13]. It is important to have discussions regarding patients’ preference in eating and drinking and make a shared decision[14], rather than making assumptions about patient perception and paternalistically making a ‘safe’ decision[15]. Issues have been raised regarding the RCP guidance on EDAR, with concerns towards the risk management approach being standardised than an evidence-based informed consent approach[6]. With EDAR guidance being published, it is our responsibility as clinicians to ensure patients’ rights are protected, while also devoting attention towards the potential barriers such as staff anxiety and knowledge[16]. Adverse events such as pneumonia or choking may be another concern when considering EDAR. While our data shows that pneumonia recurrence within 30 days was not a significant concern, previous reports have shown increased readmissions with EDAR-linked conditions such as chest infections and reduced oral intake[17]. It is important to assess which patients are appropriate for EDAR, and monitor them throughout the course through to discharge where appropriate documentation of decisions is carried through into the community.

Eating and drinking is a basic right, and decisions for or against it are not straightforward. Clinicians have the responsibility to act under the basic ethical principles of medical ethics—autonomy, beneficence, non-maleficence and justice[18]. All individuals have the freedom to eat and drink as they wish (*autonomy*). However, as it could cause harm and discomfort to the patient, clinicians provide recommendations based on the evaluated risks (*non-maleficence*), and may recommend alternative methods of nutritional intake if deemed appropriate (*beneficence).* These recommendations, however, do not always align with patient autonomy and bring forth dilemmas in the decision-making process. In addition, interventions related to dysphagia, including EDAR, are often inaccessible, leading to difficulties in maintaining equity across the community and globally (*justice*). These aspects support the importance of having guidance regarding decision-making in eating and drinking and increasing its awareness to provide a basis for all clinicians regardless of profession or setting, while additional case-based training is essential in the implementation and adaptation of EDAR and other methods in practice, as evidenced by clinical data. While EDAR is beneficial for some individuals, it is not always the best choice for individuals and caregivers, and the key lies in how to evaluate appropriate situations as a multidisciplinary team. The ethical balance between providing comfort and considering safety, or emphasising patient autonomy while being a responsible healthcare professional, is not a simple dilemma. Multidisciplinary team discussions with added expertise from stakeholders of other related specialties such as palliative care may be beneficial.

### Strengths and limitations

Some limitations must be mentioned. This study was a single-centre, retrospective study where EDAR was originally developed, and results may not translate to situations in other regions or institutions. There is a well-established dissemination route on EDAR policy and practice through robust training programmes delivered to nurses and medical staff in the developing hospital. The likelihood therefore of EDAR being initiated and utilised appropriately at the developing hospital over other institutions is higher. However, this was a relatively large study in a 521-bed hospital. There have been no similar studies of EDAR in this population. This highlights the value of this study for the next steps. This will provide a basis for addressing the complex decision-making process surrounding EDAR and what can be done to make it easier for clinicians and patients.

## Conclusion

EDAR decisions were made mostly as part of end-of-life care. EDAR should also be offered to appropriate patients in earlier disease stages, as comfort, dignity and autonomy are a priority regardless of disease stage. Underlying reasons for the low EDAR application rate must be investigated to maximise patient autonomy and comfort while minimising staff burden.

## Data Availability

All data are applicable in the paper.
